# Effectiveness of locally produced ready to use supplementary food on hemoglobin, anthropometrics, and plasma micronutrients concentrations of 6 to 23 months age children: a non-randomized community-based trial from Pakistan

**DOI:** 10.3389/fnut.2023.1176778

**Published:** 2023-07-27

**Authors:** Aslam Khan, Zia Ul-Haq, Sheraz Fazid, Sadia Fatima, Nawshad Muhammad, Jawad Ahmed, Salim Manoharadas, Sher Zaman Safi, Ijaz Habib, Cecilia Garzon, Yasir Ihtesham, Fareeda Zahid, Fazal Dad, Tanimoune Mahamadou, Nicola M. Lowe

**Affiliations:** ^1^Institute of Basic Medical Sciences, Khyber Medical University, Peshawar, Pakistan; ^2^Institute of Public Health and Social Sciences, Khyber Medical University, Peshawar, Pakistan; ^3^Institute of Health and Wellbeing, University of Glasgow, Glasgow, United Kingdom; ^4^Department of Botany and Microbiology, College of Science, King Saud University, Riyadh, Saudi Arabia; ^5^Faculty of Medicine, Bioscience and Nursing, MAHSA University, Jenjarom, Malaysia; ^6^Interdisciplinary Research Center in Biomedical Materials, COMSATS University Islamabad Lahore Campus, Lahore, Pakistan; ^7^World Food Programme, Peshawar, Pakistan; ^8^World Food Programme, Islamabad, Pakistan; ^9^UCLan Research Centre for Global Development, University of Central Lancashire, Preston, United Kingdom

**Keywords:** stunting, malnutrition, SQ-LNS, vitamin A, vitamin D, zinc, growth, anemia

## Abstract

**Background:**

Micronutrient deficiencies including vitamin A, vitamin D, and zinc are highly prevalent in children below 5 years of age in low and –middle-income countries. We aimed to evaluate the effectiveness of ready-to-use Lipid-based Nutrient Supplement—Medium Quantity (LNS-MQ) local name “Wawa-mum” on plasma micronutrient status, hemoglobin concentration and anthropometric measurements.

**Methods:**

A community-based non-randomized trial was conducted in the Kurram district of Khyber Pakhtunkhwa from January 2018 to June 2019. A total of 110 children aged 6 to 23 months old were recruited and allocated to the intervention and control arm of the study. A total of 57 children in the intervention arm received a daily ration of 50 g of Wawa-mum, for one year. To assess the impact of the intervention on primary outcome measures, i.e., serum vitamin A, D concentration, plasma zinc, and hemoglobin concentration. Blood samples were collected at baseline and after one year following the intervention. The vitamins concentration in serum were assessed using Enzyme-Linked Immunosorbent Assay (ELISA) and plasma zinc by atomic absorption spectrometry. The hemoglobin concentration was measured by an automated hematology analyzer. A 24-h dietary recall interview was used to assess the nutrient intake adequacy. Multivariate Linear regression models were used to analyze the outcomes while controlling for potential confounders.

**Results:**

In the intervention arm, children had on average 6.2 μg/dL (95% CI 3.0–9.3, value of *p*<0.001) increase in the serum vitamin A concentration, 8.1 ng/mL (95% CI 1.3–14.9, value of *p* 0.02) increase in serum vitamin D concentration and 49.0 μg/dL (95% CI 33.5–64.5, value of *p*<0.001) increase in the plasma zinc concentration, and 2.7 g/dL (95% CI 2.0–3.3, value of *p*<0.001) increase in hemoglobin concentration while adjusted for covariates. An addition, length-for-age *z*-score (LAZ), weight-for-length *z*-score (WLZ), weight-for-age *z*-score (WAZ), and prevalence of undernutrition including stunting, wasting, and underweight were calculated as a secondary outcome to investigate the impact of micronutrients on growth parameters, that has been improved significantly after receiving the Wawa-mum.

**Conclusion:**

Wawa-mum (LNS-MQ) is an effective intervention to improve the micronutrient status, hemoglobin concentration, and growth parameters in 6 to 23 months children, which can be scaled up in the existing health system to address the alarming rates of under nutrition in Pakistan and other developing countries.

**Clinical trial registration:**

https://doi.org/10.1186/ISRCTN94319790, ISRCTN94319790.

## Introduction

Nutritional status has a strong influence on child development, due to the synergistic relationships between malnutrition and illnesses (1). Nutritional deficiencies are considered to be one of the leading causes of morbidity and mortality in children under five years of age (2). There are various contributing factors to childhood under nutrition in low-income countries which are insufficient dietary intake, poor mother and child health, food insecurity, poor hygiene, insufficient consumption of dairy products, plant-based legumes (rich in protein), exclusive breastfeeding coupled with improper complementary feeding, low levels of maternal education and knowledge of nutrition, economic and social background (3, 4). Irrespective of supplementation, routine complementary feeding only may not ensure dietary adequacy for young children in low- and middle-income nations (5).

Pakistan is one of the countries with a significant burden of under nutrition, particularly among children aged 6–59 months. In Pakistan, the national nutritional Survey (NNS-2018) reported 40% stunting, 17.7% wasting, and 28.9% underweight in children below 5 years of age. Rural areas were more prone to malnutrition than urban and boys were more affected than girls. In Khyber Pakhtunkhwa Newly Merged District (NMD-KP), the prevalence of stunting, wasting, and under-weight was reported by 48.3, 23.1, and 33.7%, respectively, due to multiple natural disasters, challenges of militancy and military operations, large-scale destruction and limited access to the development of economic activities and agriculture, which reflects the worst nutrition situation among the regions of Pakistan (6). Such circumstances has the greatest effect on the most vulnerable parts of the population in terms of under nutrition, especially children under five years of age, and pregnant and lactating women, appearing as one of the major concerns.

The deficiencies of vitamin A, D, and plasma zinc are common nutritional problems that have been observed in preschool children of developing countries, especially in Southeast Asia (7). Likewise, Pakistan is facing the same problem of micronutrient deficiencies. Vitamin A and vitamin D deficiencies among children under 5 years of age were reported 51.5 and 62.7%, respectively. Similarly, the prevalence of zinc and iron deficiency anemia was reported 18.6 and 28.6%, respectively, among preschool children (6).

An adequate nutritional status is essential for a strong immune system and optimal cognitive and physical growth in the early phase of childhood (8). Researchers highlighted a need for the development of low-cost, acceptable, and effective personalized diets, in terms of their ability to mitigate against stunting and wasting (9). Various supplementary or therapeutic foods had been used or trialed such as Corn-Soy Blend Plus Plus (Super Cereal Plus, the standard additional meal provided by WFP to children aged 6 to 23 months), BP-100™, and Plumpy’Nut™ which showed low acceptance and efficacy (10, 11). It was identified to develop a ready-to-use supplementary food (RUSF) that is more acceptable, locally produced, effective, and economical than previously evaluated items. Borg *et al* (9) conducted a trial on the effectiveness of locally produced fish-based, ready-to-use supplementary food (RUSF), Corn-Soy Blend++ (CSB++), and micronutrient powders (MNP) in declining z-scores for children under 2 years in Cambodia. All three supplements were identified as equally effective in improving the growth indicators. Moreover, fortified blended food like Corn-Soy Blend has some limitations of containing many anti-nutrients, having no milk content, low concentration of micronutrients, bulky in size, and more viscous in nature. Therefore, lipid-based nutrient supplements (LNSs) which have high nutrient content and more acceptable physical texture are considered comparatively more promising supplements to be used in this context (12).

Though the findings of the already conducted studies are promising to enhance the micronutrient concentration of children, further evidence is required to evaluate the impact of locally produced ready-to-use supplementary food at the community level, particularly among those with limited resources and fragmented health care delivery system. Further, evidence on the long-term effectiveness of Wawa-mum on plasma micronutrient concentrations (Zinc, vitamin A, and vitamin D) and its relationship with anemia status and growth indicators in children aged 6–23 months at the community level, particularly in a resource-limited setting like the Pakistani context, is scarce. Studies that have already been carried out in Pakistan have solely considered the impact of Wawa-mum on children’s anthropometric outcomes and anemia status excluding plasma zinc, vitamin A, and vitamin D. The purpose of this study was to explore the effect of lipid-based nutritional supplements on micronutrient status, hemoglobin concentration along with growth parameters in 6–23 months old children, in one of the most deprived areas of Pakistan. In addition, the impact of Wawa-mum on vitamin D concentration has been investigated that is rarely reported in the literature.

## Methodology

### Study design and participants

This study is a part of “Ready to Use Supplementary Foods (RUSF) to Prevent Stunting Among Children Under Five Years in Kurram Agency,” a community-based quasi-experimental trial, undertaken from January 2018 to December 2020 in the low-resource rural areas of the tribal district Kurram of Khyber Pakhtunkhwa, Pakistan.

In Pakistan, community health services at household levels are provided through Lady Health Workers Program. One Lady Health Worker (LHW) is responsible for 100–150 households in her covered (assigned) area. In the current study, each LHW-covered area was considered an independent cluster. Furthermore, the clusters were refined using the expanded program on immunization (EPI) maps. Intervention and control clusters were selected based on natural segregation especially prominent demarcation to avoid contamination of the intervention between the study groups.

A total of 80 matched clusters were included in the main trial, 40 each in the intervention and control arm. The overall sample size of the project was 7,200 study participants from all four groups (pregnant and lactating women, 6–23 months, and 24–59 months). Among the total participants, there were 1840 children between the ages of 6 and 23 months. In the current study, the impact of Wawa-mum supplementation was investigated in a sub-sample of 110 children aged 6–23 months The trial is registered with ISRCTN ISRCTN94319790 (13).

In the current study, the impact of Wawa-mum supplementation in a sub-sample of 6–18 months old children was investigated. We included those households that had at least one child between 6–18 months old at the time of enrollment in both intervention and control arms. Households with children 6–18 months were identified using the expanded program on routine immunization (EPI) maps in the respective areas. First, a line listing of the households in the clusters was done and then systematic random sampling was used to select households containing at least one 6–18 months age child. In a third step, one eligible participant from each household was included in the trial.

### Sample size estimation

A 2-by-2 repeated measures design was adopted, with two groups of participants being measured at two different time points. The primary goal of the study was to compare the change over time in the intervention group to the change over time in the control group. Sample size were calculated for all study variables, including vitamin A, vitamin D, zinc, and hemoglobin. The maximum sample size was determined for hemoglobin; thus, the sample size estimation for this investigation was based on hemoglobin concentration. A total sample size of 94, i.e., 47 each in the intervention group and control group achieve 81% power to detect a difference in mean changes of 0.61 g/dL with SD of 1 at the first time point, an SD of 0.88 at the second time point, and correlation of 0.4 between measurement pairs. With a 20% attrition rate, the total sample size was extended to 110 children aged 6–23 months (57 in the intervention arm and 53 in the control arm). The significance level (alpha) for a two-sided, two-sample t-test was set at 0.05 (14). All these children participated in the trial after taking informed consent from their parents/guardians.

### Inclusion and exclusion criteria

**Figure 1:**
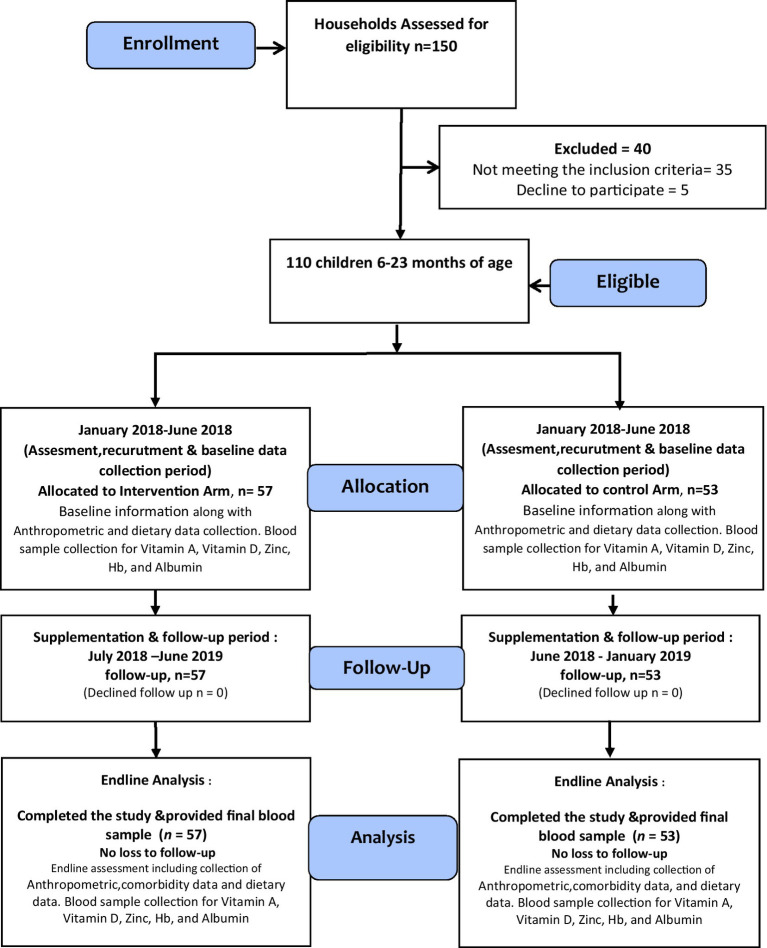
Consort diagram.

All 150 children aged 6–18 months enrolled in the study were screened for inclusion and exclusion criteria. These are apparently healthy children and not receiving other supplements. Whereas, children with severe malnutrition, i.e., (Mid Upper Arm Circumference < 11.5 cm) (MUAC), known congenital malformations identified at the baseline or severe developmental impairment such as cerebral palsy, and those with malabsorption/metabolic disorder, malignancy, with those children who were allergic to supplement ingredients, having persistent diarrhea, or who were unable to take Wawa-mum (e.g., cleft palate), and children have been regularly taking other supplements or having any known gastrointestinal tract disorders were excluded from the study (Figure 1).

### Wawa-mum intervention, composition, dosage, distribution, and utilization

Lipid-Based Nutrient Supplement Medium quantity (LNS-MQ) local name “Wawa-mum” were used as supplementary food. The active ingredients include roasted chickpeas, vegetable oil (palm, rapeseed), dry skimmed milk, sugar, vitamins & minerals, emulsifier, and antioxidants. The daily ration of Wawa-mum was one sachet of 50 g supplied to children for a period of 12 months from July 2018 to June 2019. It provided a minimum of 255 kcal of energy and most of the micronutrients according to recommended dietary allowance (RDA) (Supplementary Table S1) (15). This Wawa-mum ration was provided by UN World Food Program. These supplements were delivered by LHWs on monthly basis as a food package from the near healthcare facility for the recruited study participants in their households. There were no adverse events related to the use of these supplements were reported in the overall study. Lady Health Workers (LHW) ensured the utilization of the Wawa-mum on regular visits to all the households of the study participants to record the supplement usage, and they collected empty sachets to monitor compliance. It was ensured that the supplement was given to the targeted child on a daily basis and they encouraged.

continued use of the supplement.

### Data collection

At the baseline, demographic, socio-economic characteristics of the mothers/caregivers and dietary recall data, comorbidity, Infant and Young Child Feeding practices (IYCF) practices, vaccination status, anthropometric measurements, and blood samples of children were collected as per the standard protocol. The child’s weight and length were measured using digital weighing scales (SECA) and length measuring boards (SECA). Weight was measured in light clothing to the nearest 100 grams, while length was measured without shoes and cap to the nearest 0.1 cm, and mid-upper arm circumference (MUAC) was recorded to the nearest 1 mm (flexible UNICEF insertion tape) as per WHO standard protocol (16). Dietary intake was analyzed by trained field staff using a multiple-pass 24-h dietary recall questionnaire at two-time points, i.e., at the baseline and the end-line of the study (17). The aim of the dietary assessment was to determine the typical nutritional intake in the community only. The follow-up data were collected at the community level by a trained data collection team with the assistance of Lady Health Workers in their respective areas. At the end-line, anthropometric data, comorbid conditions of the child (diarrhea and acute respiratory diseases) in the last month, and blood samples were collected from each participant. No loss to follow-up was reported among the participant recruited for the biochemical sampling and the same participants were observed at the end-line of the trial.

### Data quality control and quality assurance

To validate the study data, a verification team (not the data collectors) randomly visited a few of the households to double-check the data accuracy. A supervisor was assigned to each cluster to keep an eye on the data enumerators and collectors. The entire data collection process was quarterly monitored by the project manager, PI, and co-PI.

### Biochemical analysis

Blood samples (non-fasting) were collected from each participant with venipuncture by a skilled phlebotomist in the community. For albumin, zinc, and vitamin A, D assessment the blood was collected in heparin tubes, trace element-free tubes and gel tubes, respectively. All samples were centrifuged to obtain plasma for zinc and albumin while serum for vitamin A, and D analysis in the pathology laboratory of DHQ hospital Parachinar Newly Merged District of Khyber Pakhtunkhwa (NMD-KPK) Pakistan. The separated plasma/serum was tightly packed in Eppendorf tubes and was transported in dry ice on a daily basis to the Biochemistry laboratory of Khyber Medical University at Peshawar KP for processing and storage at −80 C^0^ until further biochemical analysis. The plasma was used for the measurement of albumin using a fully automated chemistry analyzer (Cobas 6,000) (Roche Diagnostics). The 25(OH) vitamin D was analyzed with the ELISA technique (according to its available literature protocol) using BIOS Microwell Diagnostic System (Perkin Elmer, catalog No:10501, USA) with a cut-off value of <30 ng/mL. The vitamin A (retinol) was analyzed through an ELISA kit (Elabscience, catalog No: E-EL-0135, Wuhan China) with a cut-off value of <20 μg/dL. To measure the plasma zinc concentration, a proper wet acid digestion method was used to digest plasma and then followed by the analysis through an atomic absorption spectrometer (Model: AAS 700 Make: Perkin Elmer, USA) with a cut-off value of <65 μg/dL (18). The whole blood samples collected in EDTA-containing vacuum tubes were used to measure the hemoglobin concentration of the participant using an automated hematology analyzer (Medonic M-Series Hematology Analyzer, Sweden) with a cut-off value of <11.00 g/dL. These assays were carried out following the instructions of the manufacturer. Laboratory quality control assessment was measured with the Westgard rule on the Levey–Jennings chart (19).

### Statistical analyses

STATA version 15 was used to analyze the data. The distribution of data was checked for skewness and kurtosis for each of the micronutrient variables. All the data was normally distributed. A Chi-square test was used to assess the difference between child characteristics and study group at the baseline for categorical variables, whereas a t-test was used for continuous variables. Anthropometric indices i.e., weight-for-age (underweight), length-for-age (stunting), and weight-for-length (wasting) scores were calculated according to the WHO growth standards (20). Windiet^®^ (2005) software was used to analyze the 24-h dietary recall data for the total energy, macronutrient, and micronutrient composition of all the meals consumed. Multivariate linear regression models were used to assess the impact of LNS-MQ (Wawa-mum) on each of the plasma micronutrients, including plasma zinc, serum vitamin A, D, and hemoglobin (Hb) concentration.

A paired sample t-test was used to assess the association between the use of Wawa-mum and plasma micronutrients (Between group means differences at baseline and end-line), child anthropometric assessment, an inflammatory biomarker, and hemoglobin concentration at the end-line. Multiple linear regression models were used to assess the impact of Wawa-mum (RUSF) on each of the plasma micronutrients; plasma zinc, serum vitamin A, and vitamin D. The unadjusted model included age and study arm whereas the adjusted model additionally included child age at enrollment, gender of the child, HAZ, WHZ, WAZ score, plasma albumin status, reported diarrhea in the previous month, reported acute respiratory disease in the previous month, socioeconomic status of households (HHs), breastfeeding in the first hour when a child born, exclusive breastfeeding, complementary feeding age in months, mother age (y), mother education, mother work status, father education, father work status, energy consumed (Kcal) and baseline values of each desired outcome variable.

### Ethical consideration

Ethical approval was taken from the ethical review board of Khyber Medical University under reference No. DIR/KMU-EB/LT/000736. Each participant was informed about the study’s purpose and components. After gaining informed consent from their parents/guardians, selected children were recruited for the study.

## Results

One hundred and fifty households were considered for assessment, among which 110 households were enrolled to participate in the study. Among 40 children excluded, thirty-five were excluded due to not meeting the inclusion criteria. Five people declined to participate due to worries about blood collection and family migration to various regions. The CONSORT diagram is shown in Figure 1.

The baseline characteristics of households, caregivers, and children are shown in (Table 1). The mean age of children at baseline was (16.7 ± 4.2 months) in the intervention arm and (16.8 ± 4.7 months) in the control arm. Regarding gender distribution, more males were counted in the intervention arm 56.1% as compared to the control arm 45.2%. Breastfeeding practices were slightly better in the control arm, i.e., Breastfeeding in the first hour after birth was (77.4%) while exclusive breastfeeding up to 6 months was shown higher (89.4%) in the intervention arm. The family structure of the children in the intervention arm was comparatively more in joint nature than those in the control arm. Unemployment among the bread earner, i.e., father of the children was comparatively higher in the intervention arm than in the control. There were no noticeable differences between the arms of the study in any other demographic and anthropometric characteristics.

**Table 1:** Baseline characteristics of households, caregivers, and children by study arm.

Baseline characteristics	Intervention *N* (%)	Control *N* (%)
Socioeconomic Status of households (HHs*) *n* (%)		
Poor	36 (63.1)	33 (62.2)
Non-poor	21 (36.8)	20 (37.7)
Mother age (years)		
Mean (± SD)	30.05 (5.4)	31.49 (5.5)
Mother education		
Primary or above	10 (17.5)	7 (13.2)
No formal education	47 (82.4)	46 (86.8)
Mother work status		
Paid Work	6 (10.5)	5 (9.4)
House wife	51 (89.4)	48 (90.5)
Father education		
Primary or above	45 (78.9)	40 (75.4)
No formal education	12 (21.0)	13 (24.5)
Father work status		
Paid work	39 (68.4)	44 (83.0)
Unemployed	18 (31.5)	9 (16.9)
Family structure *n* (%)		
Single	6 (10.5)	9 (16.9)
Joint	51 (89.4)	44 (83.0)
Age of child in month (mean ± SD)	16.7 (4.2)	16.8 (4.7)
Gender of child *n* (%)		
Male	32 (56.1)	24 (45.3)
Female	25 (43.8)	29 (54.7)
Childhood specific morbidities (in the previous month)		
Diarrhea *n* (%)		
No	51 (89.5)	49 (92.4)
Yes	6 (10.5)	4 (7.5)
Acute respiratory infections *n* (%)		
No	44 (77.2)	39 (73.6)
Yes	13 (22.8)	14 (26.4)
Required vaccination status of the child *n* (%)		
Complete	47 (82.4)	45 (84.9)
Incomplete	10 (17.5)	8 (15.0)
Breastfeeding in the first hour after birth *n* (%)		
No	15 (26.3)	12 (22.6)
Yes	42 (73.7)	41 (77.3)
Exclusive breastfeeding *n* (%)		
No	6 (10.5)	7 (13.2)
Yes	51 (89.4)	46 (86.7)
Age of complementary feeding introduction (mean ± SD)		
(Mean ± SD)	6.61 (1.9)	6.54 (1.9)

^*^A household is a group of person who live under one roof and sharing the same kitchen.

Table 2 shows the impact of Wawa-mum on the plasma micronutrient status, hemoglobin (Hb) concentration, and growth parameters of children 6 to 23 months of age. On the assessment of the micronutrients from the blood samples post-intervention, there was a statistically significant increase in plasma zinc, serum vitamin A, vitamin D, and hemoglobin concentration in the intervention arm with respect to the control arm (paired sample t-test, <0.05). Within the intervention arm, the mean plasma zinc increased from 49.8 μg/dL (± 21.1 SD) to 91.8 μg/dL (±23.5 SD), this was followed by serum vitamin A, i.e., 17.3 μg/dL (±6.6 SD) to 24.8 μg/dL (±5.8 SD), serum vitamin D, i.e., 28.8 ng/mL (±1.6 SD) to 37.0 ng/mL (±1.2 SD) and hemoglobin concentration 10.2 /dL (±1.4 SD) to 12.2 g/dL (±0.6 SD) and these associations were statistically significant (value of *p* <0.05). The mean WAZ, WLZ & LAZ scores in the intervention arm increased from (mean ± SD) −1.4 (1.2), −1.4 (1.3), −0.9 (1.7) to −0.5 (0.7), −0.6 (0.8), −0.2 (1.1), respectively and were statically significant different to the control arm values (paired sample *t*-test, value of *p* <0.05). There were no significant differences in plasma albumin at baseline and end-line in both the intervention and control arm. Statistically significant increases in serum vitamin A, vitamin D, plasma zinc, and hemoglobin concentrations were observed in the intervention arm as compared to the control arm.

**Table 2:** Comparison of plasma micronutrient status, anthropometric outcomes, hemoglobin concentration, and inflammatory biomarker of children at the baseline and endline by study groups.

Characteristics	Control baseline (*N* = 53)	Control endline (*N* = 53)	Mean difference	Value of *p*	Intervention baseline (*N* = 57)	Intervention endline (*N* = 57)	Mean difference	Value of *p*
	Mean (SD)	Mean (SD)			Mean (SD)	Mean (SD)		
Growth indicators								
LAZ	−0.7 (1.3)	−0.7 (1.2)	−0.01	0.93	−0.9 (1.7)	−0.2 (1.1)	−0.7	**0.001**
WLZ	−1.5 (1.7)	−1.3 (1.1)	−0.19	0.27	−1.4 (1.3)	−0.6 (0 0.8)	−0.80	**<0.001**
WAZ	−1.3 (0.9)	−1.2 (0.7)	−0.09	0.50	−1.4 (1.2)	−0.5 (0.7)	−0.90	**<0.001**
Micronutrients status								
Plasma zinc (μg/dL)	48.8 (20.9)	57.5 (19.8)	−8.7	0.0001	49.8 (21.1)	91.8 (23.5)	−42	**<0.001**
Serum vitamin A (μg/dL)	16.4 (5.5)	17.6 (5.3)	−1.2	0.01	17.3 (6.2)	24.8 (5.8)	−7.5	**<0.001**
Serum vitamin D (ng/mL)	27.3 (1.9)	28.2 (1.3)	−0.9	0.63	28.8 (1.6)	37.0 (1.2)	−8.2	**<0.001**
Anemia status								
Anemia Hb (g/dL)	10.3 (1.3)	9.9 (1.0)	0.4	0.08	10.2 (1.4)	12.2 (0.6)	−1.9	**<0.001**
Inflammatory marker								
Total albumin (g/dL)	3.7 (0.9)	3.9 (0.6)	−0.2	0.09	3.4 (0.90)	3.5 (0.9)	−0.2	**0.35**

Analysis of the 24-h dietary recall data at baseline and end-line revealed that the average nutrient intakes of the enrolled children were lower than the recommended nutrient intakes for 6–23 months children according to the Pakistan Dietary Guidelines (21) (Supplementary Table S2). Determining the usual dietary intake in the community was the goal rather than looking into the potential effects of diet.

Multivariable linear regression showed that the intervention group (who used the Wawa-mum) had significantly higher serum concentrations of all micronutrients and hemoglobin concentration compared to the control group. The adjusted model for plasma zinc concentration revealed that children in the interventional arm had an increase of 49.0 (μg/dL) (95% CI 33.5–64.5) in the plasma zinc concentration as compared to the control group (value of *p* <0.001). The adjusted model for serum vitamin D concentration revealed that children in the intervention arm had 8.1 (ng/mL) higher serum vitamin D concentration (95% CI 1.2–14.9) and 6.2 (μg/dL) higher serum vitamin A concentration (95% CI 3.0–9.3) (value of *p* <0.05) while taking control as a reference. Similarly, the impact of Wawa-mum in the interventional arm showed an increase of 2.7 (g/dL) in hemoglobin concentration (95% CI 2.0–3.3) (Adjusted value of *p* <0.001) (Table 3).

**Table 3:** Multiple-linear regression analysis of the plasma zinc, serum vitamin A, vitamin D, and Hemoglobin concentration against nutritional outcomes and other associated factors.

Models	Results		
	Coefficient	(95% CI)	value of p
Plasma Zinc at endline of intervention group (μg/dL)			
Unadjusted	35.0	26.6, 43.4	<0.001
Adjusted^1^	49.0	33.5, 64.5	<0.001
Serum vitamin D at endline of intervention group (ng/mL)			
Unadjusted	8.8	5.3, 12.3	<0.001
Adjusted^2^	8.1	1.2, 14.9	0.02
Serum vitamin A at endline of intervention group (μg/dL)			
Unadjusted	7.3	5.1, 9.4	<0.001
Adjusted^3^	6.2	3.0, 9.3	<0.001
Hemoglobin g/dL at endline of intervention group			
Unadjusted	2.2	1.92, 2.5	<0.001
Adjusted^4^	2.7	2.06, 3.3	<0.001

^1^Model 1 = is adjusted for child age at enrollment, gender of the child, LAZ, WLZ, WAZ score, plasma albumin, reported diarrhea in the previous month, reported acute respiratory disease in the previous month, socioeconomic status of HHs, breastfeeding in the first hour when child born, exclusive breastfeeding, complementary feeding age in months, mother Age (y), mother education, mother work status, father education, father work status, vaccination status of the child, family Structure, energy consumed (Kcal) and plasma zinc concentrations at the baseline. ^2^Model 2 = is adjusted for child age at enrollment, gender of the child, LAZ, WLZ, WAZ score, reported diarrhea in the previous month, reported acute respiratory disease in the previous month, socioeconomic status of HHs, breastfeeding in the first hour when child born, exclusive breastfeeding, complementary feeding age in months, mother Age (y), mother education, mother work status, father education, father work status, vaccination status of the child, family Structure, energy consumed (Kcal) and serum vitamin D concentrations at the baseline. ^3^Model 3 = is adjusted for child age at enrollment, gender of the child, LAZ, WLZ, WAZ score, plasma albumin reported diarrhea in the previous month, reported acute respiratory disease in the previous month, socioeconomic status of HHs, breastfeeding in the first hour when child born, exclusive breastfeeding, complementary feeding age in months, mother Age (y), mother education, mother work status, father education, father work status, vaccination status of the child, family Structure, energy consumed (Kcal) and serum vitamin A concentrations at the baseline. ^4^Model 4 = is adjusted for child age at enrollment, gender of the child, LAZ, WLZ, WAZ score, plasma albumin reported diarrhea in the previous month, reported acute respiratory disease in the previous month, socioeconomic status of HHs, breastfeeding in the first hour when child born, exclusive breastfeeding, complementary feeding age in months, mother age (y), mother education, mother work status, father education, father work status, vaccination status of the child, family Structure, energy consumed (Kcal) and hemoglobin concentrations at the baseline.

Further analysis showed that the prevalence of under nutrition including stunting, wasting, and underweight was significantly reduced by 21.1, 38.6%, and 27.5%, respectively, after receiving the Wawa-mum supplementation for one year than the control group. Plasma zinc, serum vitamin AD deficiency, and anemia prevalence were reduced by 65.7, 62.6, 53.6, and 70.2%, respectively, in the intervention group (Table 4).

**Table 4:** Impact of Wawa-mum intervention on stunting, wasting and underweight, micronutrient deficiencies and anemia status in children at baseline and end-line of the trial.

Children characteristics	Control baseline *N* = 53	Intervention baseline *N* = 57	*p*-value	Control endline *N* = 53	Intervention endline *N* = 57	*P*-value
	*n* (%)	*n* (%)		*n* (%)	*n* (%)	
Nutritional status						
LAZ < -2SD	10 (18.9)	14 (24.6)	0.4	9 (17.0)	2 (3.5)	0.02
WLZ < -2SD	25 (47.2)	23 (40.4)	0.4	16 (30.2)	1 (1.8)	<0.001
WAZ < -2SD	14 (26.4)	17 (29.3)	0.7	7 (13.2)	1 (1.8)	0.02
Micronutrients status						
Plasma zinc <65 (μg/dL)	74.5	76.4	0.8	72.6	10.7	<0.001
Serum vitamin A < 20 (μg/dL)	70.6	74.6	0.6	64.7	12.0	<0.001
Serum vitamin D < 30 (ng/mL)	66.0	67.9	0.8	56.0	14.3	<0.001
Anemia status						
Anemia Hb < 11 (g/dL)	66.0	70.2	0.6	84.9	0.00	<0.001

## Discussion

This trial showed that one-year supplementation of ready-to-use lipid-based supplementary food (Wawa-mum) to 6–23 months old children significantly improve the concentration of all studied micronutrients; plasma zinc, serum vitamin D, and vitamin A along with the hemoglobin concentration in the intervention arm compared to the control arm. A significant improvement was also observed in the mean value of LAZ, WLZ, and WAZ scores after receiving the LNS-MQ supplementation versus children receiving no supplementation. Similarly, the prevalence of undernutrition including stunting, wasting, and underweight was significantly reduced after receiving the LNS-MQ supplementation as compared to the control group.

In our community-based trial, the provision of medium-quantity LNS supplementation resulted in noteworthy improvements in plasma zinc concentration in children. This study reported that the use of Wawa-mum supplementation significantly increased the mean plasma zinc concentration and reduce the prevalence of plasma zinc deficiency by 65.7%. Similar to our findings, Wessells et al., conducted a study on children 6 to 23 months of age in Burkina Faso by providing zinc supplements in either tablet (5 mg Zn) or solution form (5 mg Zn/5 mL) daily for three weeks. A considerable increase in zinc concentration was observed by providing Zn supplements in tablets and liquid compared with the placebo group (22). Lo et al., investigated the relationship between plasma zinc concentration and zinc supplements provided in iron-fortified cereal porridge and liquid multivitamin (without Zn; control group), and iron-fortified cereal porridge and multivitamin with (6 mg Zn, ZnSuppl group) and porridge fortified with zinc 6 mg/25 gram and multivitamin (without Zn, ZnFort group). The plasma zinc concentration increased following in the ZnSuppl group as compared to the control and zinc-fortified food group (23). In our study, we found a considerably higher increase in plasma zinc concentration consuming the locally produced LNS-MQ supplements” for 12 months. in comparison to the control group. This increase might be linked to the difference in supplements formulation, compliance, and duration, high consumption of the food supplements due to resource poor setting and participants having low socioeconomic background at large (24). Another study conducted by Barffour and his colleagues studied the impact of micronutrient powder (10 mg/d Zn), supplementation on children aged 6 to 23 months for 9 months. The zinc deficiency was considerably lower in the MNP group (59.1%) compared to control group (78.6%) (25). The findings of this study are in line with our results. Furthermore, the variations may be due to the non-compliance with the intervention protocol as reported by this study In contrast to our findings, Abbeddou et al. observed no effect of daily SQ-LNS supplementation containing 5 or 10 mg zinc to children aged 9 to 10 months of age on plasma zinc concentration (26). The possibility would be the absence of a significant alteration in plasma zinc concentration following the supplementation of an SQ-LNS (ready-to-use lipid-based nutrient supplements) indicates potential challenges with inadequate absorption of supplementary zinc when taken with food, or potential variations in post-absorptive zinc metabolism.

Another finding of our trial was the substantial increase in the mean vitamin A concentration and reduction in the prevalence of vitamin A after Wawa-mum (LNS-MQ) supplementation in the intervention arm vs. the control arm. Similar to our study, Oliveira et al., evaluated the effect of multiple micronutrient powders for 2 months of supplementations on the serum vitamin A concentration of children aged 11 to 14 months. The serum vitamin A deficiency was found significantly less prevalent in the intervention group (4.7%) compared to the control group (18·6%) (27).

In contrast to our findings, Haskell et al., studied the impact of SQ-LNS on serum vitamin A in children aged 6 to 18 months in Malawia and Ghana but did not find any change in serum vitamin A concentration. The authors attribute this lack of effect due to the low prevalence of VAD in the population and poor sensitivity to changes in the status of serum retinol concentration (28).

To our knowledge, the results of this trial provide the initial evidence on the effects of locally produced lipid-based nutrient supplements (Wawa-mum) on the status of serum vitamin D, which is rarely reported in the literature. In our trial, we found a significant increase in the mean serum 25-hydroxy vitamin D [25(OH) vitamin D] concentration and a significant reduction in the prevalence of vitamin D deficiency (53.5%) after supplementation in comparison to the control group (10% reduction). Brett et al., conducted a study on 25(OH) vitamin D fortified dairy products provided for 12 weeks to healthy children aged 2 to 8 years in the Montreal region of Canada. The children were assigned to one of three groups, i.e., Control, EAR, and RDA. At baseline, all the group has vitamin D concentration of 58 nmol/L which increased to 65 nmol/L after 12 weeks of vitamin D supplementation in EAR and RDA groups compared to the control group having 55 nmol/L (29). Similar to our findings, Unger, SA. et al., investigated the effect of micronutrient fortification of lipid-based supplements (SQ-LNS) on serum 25(OH) vitamin D concentration in children under 5 years of age provided for 6 and 12 weeks. The study reported a 12% decrease in serum vitamin D deficiency in the fortified SQ-LNS group relative to unfortified SQ-LNS and there was a dose–response relationship as well (30). Marjolijn D et al., also reported a significant mean increase in serum vitamin D concentration and a decrease in the prevalence of vitamin D deficiency in 1–3 years children after supplementation of micronutrient-fortified young-child formula (YCF) versus unfortified cow milk. In this study, our findings of considerable reduction in vitamin D deficiency might be attributed to the long duration of supplementation, composition, dosage, food vehicle, and compliance of supplements. In contrast, Another study conducted by Ahmad et al., reported no improvement in concentration of 25(OH) vitamin D with supplementation of LNS for one month (31). This lack of impact might be due to different age groups and the short duration of exposure to the intervention.

Our trial demonstrated that Wawa-mum (LNS-MQ) supplementation was effective in improving the mean hemoglobin concentration in the intervention arm and also significantly reducing anemia prevalence by 72.2%. The findings of this study are consistent with several other studies on the effects of LNS supplementation on anemia (32, 33). Stewart et al., studied the impact of LNS in infants less than 12 months and found 40% reduction in in the prevalence of anemia. A similar study was conducted by Smuts et al., and found a significant increase in hemoglobin concentration and anemia deficiency (34). Khan et al., showed a 3% reduction in the prevalence of anemia (91% vs. 94%) in the intervention group as compared to the control group after the administration of the concerned supplements (35). This study reported that there was food sharing with other household members and it might have reduced the impact of the supplements on reducing anemia in the study population. In Bangladesh, LNS supplements were provided to 18 months old children, and observed a significant increase in hemoglobin concentration (36). A study conducted at Dhaka on hemoglobin concentration of children aged 6 to 23 months with supplementation (4 months) of micronutrient powder (MNP) showed a significant increase in hemoglobin concentration (37). While Contrary to our findings, Rosado et al. did not observe any significant difference among the three study groups [powdered milk (PM)], Oportunidades food supplement OFS and placebo (PL) in the reduction of anemia in children aged 12–24 months old for 6 months. However, the prevalence of anemia was reduced among all the groups and it was highlighted that this reduction might be attributed to the routine diet of the study participants (38). Other than the effect of diet it might be the composition of the supplements, the baseline nutritional status of the target population, the period of the intervention, and other factors (health surveillance, economic benefits, or education, are also important to improve Hb) responsible for anemia (39). The consistency of our results to other studies on the effect of LNS-MQ on anemia revealed that these results are broadly generalizable to the population where anemia and micronutrients deficiency are the main public concern.

In our study, we also reported a significant impact of Wawa-mum (LNS-MQ) on child growth indicators, i.e., LAZ, WLZ, and WAZ scores with a significant reduction in the prevalence of under nutrition stunting (21.1%,), wasting (38.6%), and underweight (27.5%) respectively after one year of supplementation. A review reported by Salam et al., concluded that the LNS administered to 6–23 month children significantly reduce the prevalence of stunting, wasting and underweight in the intervention group as compared to the control group (32). Another study conducted by Ying Yang Bao, a soya bean-based supplement was provided to children less than 2 years in a backward area of China. This study found a significant decrease in the prevalence of stunting, wasting, and underweight (40). In Pakistan a study conducted by Khan et al., in Thatta, Sindh Province, Pakistan where the LNS-MQ supplements were provided to children aged 6 to 23 months and found a significant reduction in stunting (9%) and wasting (22%) except under-weight (35). As compared to these studies, we found a higher percent reduction in growth indicators deficiency in the intervention group which could be attributed to differences in study design, context, duration of supplementation, acceptability of the supplement Wawa-mum, and individual characteristics which can potentially modify the impact on child outcomes. Similar studies were conducted in Bangladesh, Haiti, Peru, and Malawi that showed a decrease in the prevalence of stunting wasting, and underweight with various supplementations (41–44). Contrary to our results Borg et al., tested the effectiveness of fish-based RUSF on Cambodian infants to prevent growth faltering. The RUSF group did not differ significantly from the control for WAZ, LAZ, and WLZ (in other words, WAZ and LAZ decreased and WLZ did not change), but had increased MUAC in comparison to the control group (45). This lack of impact of the intervention was attributed to less absorption of nutrients due to high prevalence of diarrhea, replacement of breastmilk and usual consumption of food and gender of the child. While in a study conducted by Maleta et al., did not find the effect of LNS supplementation on the growth parameters (46) which has been assumed as a result of infections, environmental enteropathy, and unfavorable intestinal bacterial microbiota that determine the nutritional status of a child’s (47, 48).

We observed no significant change in plasma albumin in both arms from baseline to end-line during the trial. However, this biomarker was found in normal ranges at baseline and end-line in both control and intervention group. Plasma albumin is an acute phase reactant and is influenced by both acute and chronic inflammation which is frequently present alongside micronutrient deficiencies (49).In our trial, the normal concentration of albumin shows that the assessed concentration of the plasma micronutrients reflect the actual nutritional status rather than an altered response to inflammation. The average macro and micronutrient intake in 24-h recall data were measured as lower than the recommended dietary intake in light of the Pakistan Dietary Guideline for Better Nutrition recommendations (21). Given the deprivation and low socioeconomic conditions of the study area, it is difficult to provide children with the necessary food for their healthy development.

This one-year follow-up study assessed the impact of locally produced lipid-based nutritional supplements (LNS-MQ) on plasma micronutrient concentrations, hemoglobin concentration, and growth indicators of children between the age of 6 and 23 months at the community level. This is the first study in the erstwhile Federally Administrative Tribal Area (FATA) that assesses the impact of Wawa-mum (LNS-MQ) on plasma micronutrient status at the community level.

In conclusion, Wawa-mum (LNS-MQ) is an effective intervention to improve the micronutrient concentration, especially plasma zinc, serum vitamin A, and vitamin D concentration in children. It is also effective in improving hemoglobin concentration which is one of the key biomarkers for child nutritional anemia. A significant effect of Wawa-mum was also observed on the improvement of LAZ, WLZ, and WAZ scores. Findings from this trial are relatively new evidence regarding the impact of Wawa-mum on micronutrient status including vitamin D, which was not reported earlier. The significant impact of Wawa-mum on micronutrient deficiencies, nutritional anemia, and growth indicators is the key to addressing the alarming rates of under nutrition in Pakistan and other developing countries at an affordable cost.

### Strength and limitation

The strength of this study is that it is a community-based trial that was successfully completed. There was an uninterrupted supply of Wawa-mum to the study participants. The intervention package was delivered through the existing government-supported LHW program. We made every effort to assure data quality. No issue in compliance was reported which result in no loss to follow-up over the study course. We also assessed inflammation status among the study participants to ensure that the assessed concentration of the micronutrients reflect the actual nutritional status rather than an altered response to inflammation. Sharing of supplements and food is a common practice in local communities, however, Wawa-mum intake was closely monitored by Lady Health Workers (LHW) which ensured that the supplements were consumed by the study participant. Limitations of this study include; random allocation which is the gold standard in interventional studies was not applied however to avoid intervention contamination between study groups, intervention and control groups were chosen based on natural segregation. Dietary assessment was conducted using a single 24-h dietary recall. May not reflect the usual micronutrient intake of the children. However, the intent was to evaluate average group-level intake. Considering the variations among sensitivity, specificity, and reproducibility for the ELISA method, HPLC would be preferred. Hence the use of the ELISA method would be a limitation of this study. Even though it was a readily available and cost-effective and rapid testing technique with reliable measurements. C-reactive protein (CRP) and alpha-1-glycoprotein (AGP) are acute-phase proteins that increase rapidly in response to inflammation for acute and chronic inflammation instead of serum albumin which has not been measured in this study. However, albumin could be influenced by inflammation and hence be a marker for nutritional status. Despite these limitations, our results provide valuable information about LNS-MQ (Wawa-mum) community-based intervention in a region, which has experienced protracted security threats, mass internal population displacement, poor food security, and limited access to quality health services.

## Data availability statement

The original contributions presented in the study are included in the article/Supplementary material, further inquiries can be directed to the corresponding author.

## Ethics statement

The studies involving human participants were reviewed and approved by Khyber Medical University Ethics board (KMU-Ethics Board) reference No: DIR/KMU-EB/LT/000736. Written informed consent to participate in this study was provided by the participants’ legal guardian/next of kin.

## Author contributions

AK: experimental and project design, conceptualization, sample collection and analysis, original draft write-up, and writing–review and editing. ZU-H: supervision and project acquisition. ShF: data analysis. SaF: data review. NM: polish article write up. JA: review and improve the article write up. IH: technical assistance in data collection, technical advisor & budget development, micro planning and supply chain management. CG and YI: provide funding, project review, overall strategic direction and project acquisition. FZ: technical assistance in data collection. FD: food distribution. TM: review of the project, review of data, technical advisor and fund arrangement from donors. NL: critical review of the article and arrangement of data presentation. SM and SS: critical review of the manuscript and checking of fluency and of english grammar. All authors contributed to the article and approved the submitted version.

## Funding

This study was fully funded by UN-WFP Pakistan. They also ensured the timely provision of all the nutritional supplements throughout the project. Also, they provided monitoring support throughout the project. We would also like to acknowledge the Researchers Supporting Project, number (RSPD2023 R708), King Saud University, Riyadh, Saudi Arabia.

## Conflict of interest

The authors declare that the research was conducted in the absence of any commercial or financial relationships that could be construed as a potential conflict of interest.

## Publisher’s note

All claims expressed in this article are solely those of the authors and do not necessarily represent those of their affiliated organizations, or those of the publisher, the editors and the reviewers. Any product that may be evaluated in this article, or claim that may be made by its manufacturer, is not guaranteed or endorsed by the publisher.
