# Supplementary material for: Effectiveness of locally produced ready to use supplementary food on hemoglobin, anthropometrics, and plasma micronutrients concentrations of 6 to 23 months age children: a non-randomized community-based trial from Pakistan
Source: Front Nutr. 2023 Jul 27;10:1176778. doi: 10.3389/fnut.2023.1176778 (PMC10415027; doi:10.3389/fnut.2023.1176778)
Supplement: Supplementary file 1 [file Data_Sheet_1.pdf]

Table S1. Composition of nutrient values in LNS-MQ supplement (Wawa-mum).

**Nutrients values per 50 g product (one serving)**	**Unit**	**Minimum**	**Maximum**	**Recommended daily allowances for the Pakistani population for 6 to 23 months children**
Energy	kcal	255	280	820 -1250
Protein	g	5.5	8	12-23
Fat	g	13	18	
ω-3 fatty acids	g	0.15	0.9	0.5-0.7
ω-6 fatty acid	g	1.3	3.1	4.6-7
Retinol (VitaminA)	mcg	275	575	350-400
Thiamin (VitaminB1)	mg	0.5	-	0.3-0.5
Riboflavin (Vitamin B2)	mg	1.05	-	0.4-0.5
Niacin (VitaminB3)	mg	6.5	-	4-6
Pantothenic Acid (Vitamin B5)	mg	2	-	1.8-2
Pyridoxine (Vitamin B6)	mg	0.9	-	0.3-0.5
Biotin (VitaminB7)	mcg	30	-	6-8
Folate (VitaminB9)	mcg	165	-	80-150
Cobalamin (Vitamin B12)	mcg	1	-	0.5-0.9
Ascorbate (VitaminC)	mg	30	-	50-15
Cholecalciferol (Vitamin D)	mcg	7.5	10	10
Tocopherol Acetate (Vitamin E)	mg aTE	8	-	5-6
Phytomenadione (Vitamin K)	mcg	13.5	-	2.5-30
Calcium (Ca)	mg	268	375	260-700
Copper (Cu)	mg	0.7	1.0	220-340
Iodine (I)	mcg	50	70	50-90
Iron (Fe)	mg	5	7	15-20
Magnesium (Mg)	mg	75	113	75-80
Manganese (Mn)	mg	0.6	1.2	0.6-1.2
Phosphorus (P)	mg	225	375	275-460
Potassium (K)	mg	450	700	0.7-3.0
Selenium (Se)	mcg	10	20	20
Sodium (Na)	mg	-	135	0.37-1.0
Zinc (Zn)	mg	5.5	7	15

Table S2: Dietary intake at baseline and endline

Variable	Control baseline (N=53)	Control endline (N=53)	P-value	Intervention baseline N=57	Intervention endline (N=57)	P-value
	**Mean (SD)**	**Mean (SD)**		**Mean (SD)**	**Mean (SD)**	
Energy (kcal/d)	849.8 (97)	1033.2(114)	**<0.001**	832.6(110)	1091.2(141)	**<0.001**
Fat (g/d)	17.50 (9.4)	25.3(11.3)	**<0.001**	15.8(8.6)	26.4(11.0)	**<0.001**
Carbohydrate (g/d)	130.18(37.2)	137.1(42.5)	**0.3**	129.8 (35.3)	148.8(43.5)	**0.002**
Protein (g/d)	11.7(1.4)	18.7(1.8)	**<0.001**	11.5(2.13)	19.7(7.4)	**<0.001**
Vitamin A (µg/d)	185.80(63)	297(49)	**<0.001**	178.2(49)	312(131.4)	**<0.001**
Vitamin D (µg/d)	0.5(0.5)	1.50(0.4)	**<0.001**	0.7(0.6)	1.9(0.5)	**<0.001**
Iron (mg/d)	4.6(1.2)	10.0(0.8)	**<0.001**	4.5 (1.5)	9.7(2.0)	**<0.001**
Zinc (mg/d)	3.0 (0.8)	6.6 (1.13)	**<0.001**	3.50 (1.2)	8.0(1.8)	**<0.001**

Table S3: Multiple-linear regression analysis of the plasma zinc, serum vitamin A, vitamin D, and Hemoglobin concentration against nutritional outcomes and other associated factors.

**Models**	**Results**		
**Plasma Zinc (µg/dL) at endline of intervention group**	**Coefficient**	**(95% Conf. Interval)**	**P-value**
**Unadjusted model:**	35.05	26.61,43.49	<0.001
**Adjusted Model:**			
**Age baseline**	0.47	-0.61 , 1.5	0.38
**Gender of child**			
Female	3.80	-5.98 , 13.58	0.44
Male	Reference		
**Study Arm**			
Intervention	**49.0**	**33.5 , 64.5**	**< 0.001**
Control	Reference		
**LAZ** <-2SD	-62.39	-142.82 ,18.04	0.12
**WLZ** <-2SD	-52.46	-119.24 , 14.32	0.12
**WAZ** <-2SD	86.07	-30.55 , 202.71	0.14
**Plasma zinc (µg/dL) at baseline**	0.42	0.20 , 0.64	< 0.001
**Diarrhea reported at endline**	0.87	-11.80 , 13.53	0.89
**Respiratory disease reported at endline**	0.09	-10.19 , 10.37	0.98

**Energy (Kcal)at endline**	-0.04	- 0.07 , -0.00	0.02
**Mother Education**	3.61	-6.10 , 13.34	0.46
**Mother Work status**	0.96	-14.02 ,15.95	0.89
**Father Education**	1.87	-8.95 , 12.69	0.73
**Father Work Status**	5.64	-4.38 , 15.67	0.26
**Family Structure**	3.41	-8.94 , 15.78	0.58
**Breastfeeding in the first hour after birth**	2.33	-8.27 , 12.93	0.66
**Exclusive Breastfeeding**	-7.22	-16.42 , 1.98	0.12
**Required Vaccination status of the child**	3.35	-6.57 , 13.28	0.50
**Age of complementary feeding introduction (mean± SD)**	0.82	-1.48 , 3.13	0.48
**Mother Age (years)**	0.16	-0.75 , 1.09	0.71
**Poverty Score**	-0.88	-10.41 , 8.64	0.85
**Serum vitamin D (ng/ml) at endline of intervention group**			
**Unadjusted model:**	**8.84**	**5.31, 12.37**	**<0.001**
**Adjusted model:**			
**Age baseline**	-0.16	-0.63 , 0.30	0.48
**Gender of child**			
Female	-0.77	-4.80 , 3.25	0.70
Male	Reference		
**Study Arm**			
Intervention	**8.11**	**1.2 , 14.9**	**0.02**
Control	Reference		
**LAZ** <-2SD	-16.91	-51.74 , 17.91	0.33
**WLZ** <-2SD	-13.40	-42.34 , 15.52	0.35
**WAZ** <-2SD	25.72	-24.74 , 76.19	0.31
**Serum vitamin D (ng/mL) at baseline**	0.22	0.06 , 0.38	< 0.001
**Diarrhea reported at endline**	-0.18	-5.62 , 5.24	0.94
**Respiratory disease reported at endline**	2.06	-2.39 , 6.52	0.35
**Energy (Kcal)at endline**	-0.00	-0.02 , 0.01	0.58
**Mother Education**	3.21	-1.05 , 7.47	0.13
**Mother Work status**	-1.12	-7.25 , 5.00	0.71
**Father Education**	1.14	-3.43 , 5.73	0.62
**Father Work Status**	0.33	-3.98 , 4.64	0.87
**Family Structure**	1.46	-3.83 , 6.76	0.58
**Breastfeeding in the first hour after birth**	0.76	-3.73 , 5.27	0.73
**Exclusive Breastfeeding**	-0.90	-4.80 , 2.99	0.64
**Required Vaccination status of the child**	-3.87	-8.11 , 0.35	0.07
**Age of complementary feeding introduction (mean± SD)**	1.05	0.07 , 2.04	0.03
**Mother Age (years)**	0.00	-0.39 , 0.41	0.96
**Poverty Score**	-3.75	-7.80 , 0.29	0.06
**Seum vitamin A** (µg/dL) **at endline of intervention group**			
**Unadjusted model:**	**7.30**	**5.11, 9.48**	**<0.001**
**Adjusted model:**			
**Age baseline**	0.29	0.08 , 0.49	0.00
**Gender of child**			
Female	0.627	-1.08 , 2.34	0.46
Male	Reference		
**Study Arm**			
Intervention	**6.2**	**3.0 , 9.3**	**< 0.001**
Control	Reference		
**LAZ** <-2SD	-7.82	-23.00 , 7.34	0.30
**WLZ** <-2SD	-6.16	-18.78 , 6.45	0.33
**WAZ** <-2SD	11.55	-10.41 , 33.52	0.29
**Plasma vitamin A (µg/dL) at baseline**	0.69	0.54 , 0.85	< 0.001
**Diarrhea reported at endline**	0.91	-1.34 , 3.17	0.420
**Respiratory disease reported at endline**	0.87	-1.07 , 2.82	0.37
**Energy (Kcal) at endline**	0.000	-0.00 , 0.00	0.91

**Mother Education**	-1.49	-3.27 , 0.28	0.09
**Mother Work status**	0.59	-1.97 , 3.15	0.64
**Father Education**	-0.96	-2.97 , 1.04	0.34
**Father Work Status**	0.50	-1.41 , 2.41	0.60
**Family Structure**	-0.40	-2.68 , 1.86	0.72
**Breastfeeding in the first hour after birth**	0.26	-1.75 , 2.28	0.79
**Exclusive Breastfeeding**	-0.02	-1.68 , 1.62	0.97
**Required vaccination status of the child**	1.14	-0.70 , 3.00	0.22
**Age of complementary feeding introduction (mean± SD)**	0.26	-0.16 , 0.70	0.21
**Mother Age (years)**	-0.003	-0.18 , 0.17	0.97
**Poverty Score**	0.52	-1.23 , 2.28	0.55
**Anemia (Hb g/dl) at endline of intervention group**			
**Unadjusted model:**	**2.24**	**1.92, 2.55**	**<0.001**
**Adjusted model:**			
**Age baseline**	-0.00	-0.05 , 0.036	0.72
**Gender of child**			
Female	0.12	-0.25 , 0.50	0.50
Male	Reference		
**Study Arm**			
Intervention	**2.6**	**2.0 , 3.3**	**< 0.001**
Control	Reference		
**LAZ** <-2SD	-0.10	-3.34 , 3.13	0.94
**WLZ** <-2SD	-0.04	-2.74 , 2.65	0.97
**WAZ** <-2SD	-0.08	-4.79 , 4.62	0.97
**Anemia Hb (g/dL) at baseline**	0.06	-0.07 , 0.19	0.37
**Diarrhea reported at endline**	0.01	-0.46 , 0.50	0.94
**Respiratory disease reported at endline**	0.07	-0.33 , 0.48	0.70
**Energy (Kcal)at endline**	-0.000	-0.002 , 0.00	0.29
**Mother Education**	0.01	-0.37 , 0.40	0.93
**Mother Work status**	-0.20	-0.78 , 0.36	0.47
**Father Education**	-0.09	-0.53 , 0.33	0.65
**Father Work Status**	-0.06	-0.47 , 0.34	0.75
**Family Structure**	-0.39	-0.89 , 0.10	0.12
**Breastfeeding in the first hour after birth**	-0.13	-0.55 , 0.29	0.53
**Exclusive Breastfeeding**	-0.17	-0.53 , 0.18	0.32
**Required vaccination status of the child**	-0.01	-0.41 , 0.38	0.93
**Age of complementary feeding introduction (mean± SD)**	0.09	-0.00 , 0.18	0.06
**Mother Age (years)**	0.00	-0.03 , 0.03	0.93
**Poverty Score**	0.04	-0.33 , 0.43	0.79
